# Influenza hospitalizations during childhood in children born preterm

**DOI:** 10.1111/irv.12908

**Published:** 2021-09-14

**Authors:** Siri H. Hauge, Birgitte Freiesleben de Blasio, Siri E. Håberg, Laura Oakley

**Affiliations:** ^1^ Division of Infection Control and Environmental Health Norwegian Institute of Public Health Oslo Norway; ^2^ Oslo Centre for Biostatistics and Epidemiology, Department of Biostatistics, Institute of Basic Medical Sciences University of Oslo Oslo Norway; ^3^ Centre for Fertility and Health Norwegian Institute of Public Health Oslo Norway; ^4^ Department of Epidemiology and Population Health London School of Hygiene and Tropical Medicine London UK

**Keywords:** hospitalization, infant premature, influenza human

## Abstract

**Objective:**

The objective is to determine if children born preterm were at increased risk of influenza hospitalization up to age five.

**Methods:**

National registry data on all children born in Norway between 2008 and 2011 were used in Cox regression models to estimate adjusted hazard ratios (aHRs) for influenza hospitalizations up to age five in children born preterm (<37 pregnancy weeks). HRs were also estimated separately for very preterm (<32 weeks), early term (37–38 weeks), and post‐term (≥42 weeks) children.

**Results:**

Among 238,628 children born in Norway from January 2008 to December 2011, 15,086 (6.3%) were born preterm. There were 754 (0.3%) children hospitalized with influenza before age five. The rate of hospitalizations in children born preterm was 13.8 per 10,000 person‐years (95% confidence interval [CI] [11.3, 16.7]), and 5.9 per 10,000 person‐years (95% CI [5.5, 6.4]) in children born at term (≥37 weeks). Children born preterm had a higher risk of influenza hospitalization before age 5: aHR 2.33 (95% CI [1.85, 2.93]). The risk increased with decreasing gestational age and was highest among those born extremely/very preterm; aHR 4.07 (95% CI [2.63, 6.31]). Compared with children born at 40–41 weeks, children born early term also had an elevated risk of influenza hospitalization; aHR (37 weeks) 1.89 (95% CI [1.43, 2.50]), aHR (38 weeks) 1.43 (95% CI [1.15, 1.78]).

**Conclusion:**

Children born preterm had a higher risk of influenza hospitalizations before age five. An elevated risk was also present among children born at an early term. Children born preterm could benefit from influenza vaccinations.

## 1 INTRODUCTION

Annual influenza outbreaks cause severe and fatal infections in children worldwide, and children are at higher risk of influenza hospitalization compared with many other age groups.^1^, ^2^ To reduce the risk of severe influenza disease and influenza deaths, the World Health Organization (WHO) recommends risk groups to receive an annual influenza vaccine. Both the WHO and the US Centre for Disease Control (CDC) include children younger than 5 years as a priority group for influenza vaccination; however, being preterm is not recognized as an additional risk factor.^3^, ^4^ In tropical and subtropical countries, national guidelines for prioritizing influenza vaccines are often absent.^5^ In Norway, children in general are not included in the annual influenza vaccination programme. Only children with risk conditions, such as lung and heart disease, diabetes, neurological conditions, renal or liver failure or immuno‐compromised disorders are recommended the annual influenza vaccination. Being born preterm is not identified as a risk condition in Norway.^6^

The WHO defines preterm birth as children born before 37 completed weeks of gestation, and globally more than 10% of live births, around 15 million children every year, are estimated to be born preterm.^7^, ^8^ These births are associated with substantial morbidity, and one million deaths a year have been attributed preterm birth.^9^ There is a disproportionately higher burden of preterm births in low‐ and middle‐income countries. High‐income countries tend to have lower rates, though in some high‐income countries, such as Austria and the United States, the proportion of preterm births is higher than 10%.^10^, ^11^ Norway has one of the lowest preterm birth rates in the world (5.6%).^12^ Established maternal risk factors for preterm birth include African‐American/African‐Caribbean ethnicity, low or high maternal age, multiple pregnancy, maternal infections and low socioeconomic status.^13^ Improved treatment has increased the chance of survival in preterm infants, particularly for those born extremely preterm (before 28 weeks).^14^, ^15^ Increased survival, combined with a continuing high—and for some increasing—proportion of preterm births in many countries, has led to an growing number of children in the population who were born preterm.^10^, ^16^, ^17^

For all children, and especially those born preterm, respiratory tract infections cause a substantial number of hospitalizations and fatalities during the first years of life.^9^, ^18^, ^19^, ^20^, ^21^ Children born preterm have higher overall morbidity and mortality^22^ and an increased risk of hospitalizations due to infections.^23^, ^24^ Previous studies focusing on preterm birth as a potential risk factor for infections have mostly focused on hospitalizations with respiratory infections overall or respiratory syncytial virus (RSV) infections, and not on influenza.^18^, ^25^, ^26^, ^27^, ^28^ There is a scarcity of studies investigating influenza hospitalizations among preterm children beyond infancy, and currently insufficient evidence to implement vaccine recommendations for this group. In addition, it is not known to what extent risk differs with different gestational lengths, and whether children born early term also may be at higher risk.

Our aim was to assess whether children born preterm or early term were at increased risk of being hospitalized with influenza in the first 5 years of life. By including being born preterm as a risk factor for severe influenza beyond infancy could inform decisions on including these children in annual influenza vaccination programmes around the world.

## 2 METHODS

We included all children born in Norway between 1 January 2008 and 31 December 2011, and followed them to age five. We used data from two national health registries in Norway: the Medical Birth Registry of Norway (MBRN)^29^ and the Norwegian Patient Registry (NPR).^30^, ^31^ The MBRN contains individual data on all births in Norway, and we used information on gestational age at birth, sex, season of birth, multiple births, maternal age at delivery, parity and maternal smoking in pregnancy. Parental educational status was obtained from Statistics Norway and categorized according to the highest completed academic level for either parent in 2013. The NPR holds data on ICD‐10 diagnoses for all hospitalizations in Norway, and reporting to NPR is mandatory. From the NPR, we collected data on hospital admissions with an influenza diagnosis for all children in the first 5 years of life. Data were linked across registries using Norwegian personal identification numbers.

### 2.1 Gestational age

Completed gestational weeks at birth are recorded by the MBRN and based on routine ultrasound measurements when these were available (for 98% of the children), or last menstrual period when ultrasound estimations were missing. We categorized gestational age at birth into term (≥37 completed weeks) or preterm (<37 weeks), with the latter category additionally subdivided into extremely/very preterm (<32 weeks) and moderately/late preterm (32 to <37 weeks). The reference category was term births (≥37 weeks). To assess potential risks within the ‘term’ category, gestational age was further divided into eight categories (<33, 33–34, 35–36, 37, 38, 39, 40–41, ≥42), with 40–41 weeks as the reference category.

### 2.2 Influenza hospitalizations

An influenza hospitalization was defined as any hospitalization recorded on the NPR with the codes J09 (‘Influenza due to identified zoonotic or pandemic influenza virus’), J10 (‘Influenza due to identified seasonal influenza virus’) or J11 (‘Influenza, virus not identified’) as listed in the 10th revision of the International Statistical Classification of Diseases (ICD‐10). In ICD‐10, laboratory confirmation is required for the J09 and the J10 diagnoses, but not for the J11 diagnosis. To reduce potential misclassification, we excluded hospital admissions outside the yearly influenza surveillance period in Norway (running from October to May). Details of data sources and categorizations are presented in Table S1.

We used Cox regression modelling to assess associations between gestational age at birth and hospital admission for influenza up to age 5, using age in days from birth as the underlying time metric. Children were followed from birth until the first influenza hospitalization, death, emigration, or their fifth birthday. In additional analyses, we estimated the risk of influenza hospitalization in three different age groups (<1 year, 1 year and 2–4 years). A sensitivity analysis excluded multiple births. As potential confounders, we included child sex, maternal parity, maternal age, multiple birth, season of birth, parental education and maternal smoking. We conducted complete case analyses and adjusted for these covariates in our models. Dependency between siblings was taken into account by the use of robust standard errors. The proportional hazards assumption was evaluated by visual inspection of cumulative hazard curves and by testing Schoenfeld residuals. Analysis was performed using Stata 15 (StataCorp. 2017. Stata Statistical Software: Release 15. College Station, TX: StataCorp LLC).

The Norwegian Regional Committee for Medicine and Health Research Ethics approved this study and provided a waiver of consent for participants.

## 3 RESULTS

There were 245,281 children registered in the birth registry of Norway between 1 January 2008 and 31 December 2011. Less than 3% were excluded due to missing covariate information (*n* = 4171) or invalid linkage (*n* = 2482), leaving 238,628 children for analyses (Figure S1). In total, 15,086 (6.3%) of the children were born preterm (<37 weeks). Of these, 12,941 (85.8%) were moderate/late preterm (born between Weeks 32 and 36), and 2145 (14.2%) were extremely/very preterm (born before 32 weeks gestation). Preterm children were more likely to be multiple births, male, first‐born, and have a younger or older mother, and a mother who smoked during pregnancy; and less likely to have at least one parent with college or university education (Table 1).

**TABLE 1:** Perinatal and child characteristics of children born between 1 January 2008 and 31 December 2011 in Norway

	All children	Preterm (<37 weeks)		Influenza hospitalization <5 years	
	*n*	*n*	(%)	*n*	(%)
All children	238,628	15,086	(6.3)	754	(0.3)
Maternal age at birth					
<20	5208	437	(8.4)	23	(0.4)
20–24	34,672	2272	(6.6)	99	(0.3)
25–29	74,101	4287	(5.8)	224	(0.3)
30–34	77,697	4587	(5.9)	251	(0.3)
35–39	39,416	2802	(7.1)	137	(0.3)
≥40	7534	701	(9.3)	20	(0.3)
Maternal smoking in pregnancy					
No	179,883	10,954	(6.1)	536	(0.3)
Yes	24,496	1930	(7.9)	106	(0.4)
Information declined	34,249	2202	(6.4)	112	(0.3)
Child sex					
Male	122,749	8083	(6.6)	447	(0.4)
Female	115,879	7003	(6.0)	307	(0.3)
Birth order					
1	101,747	7374	(7.2)	258	(0.3)
≥2	136,881	7712	(5.6)	496	(0.4)
Multiple birth					
Yes	7872	3758	(47.7)	37	(0.5)
No	230,756	11,328	(4.9)	717	(0.3)
Parental college/university education					
No	95,780	6697	(7.0)	352	(0.5)
Yes	142,848	8389	(5.9)	402	(0.2)
Season of birth					
Winter	54,943	3741	(6.8)	158	(0.3)
Spring	61,212	3783	(6.2)	178	(0.3)
Summer	63,979	4018	(6.3)	201	(0.3)
Autumn	58,494	3544	(6.1)	217	(0.3)

**FIGURE 1:**
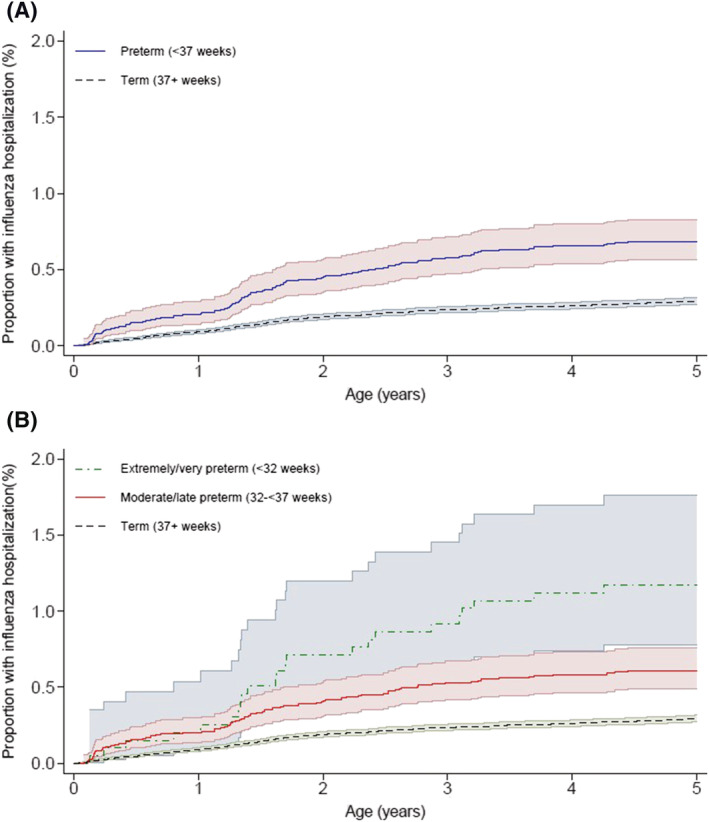
Cumulative incidence of influenza hospitalization to age 5, by (A) preterm status (preterm, term) and (B) preterm group (extremely/very preterm, moderate/late preterm and term)

Overall, 754 (0.3%) children were hospitalized with influenza below the age of five (Table S2). Of these, 101 (13.4%) were born preterm. Compared with children born at term, the cumulative incidence of hospital admission with influenza was higher in children born preterm (Figure 1A), and even higher among very preterm infants (Figure 1B). The rate in preterm children was 13.8 per 10,000 person‐years (95% confidence interval [CI] [11.3, 16.7]), whereas in term children, the rate was 5.9 per 10,000 person‐years (95% CI [5.5, 6.4]). Compared with term children, the adjusted hazard ratio for hospitalization before age five in children born preterm was 2.33 (95% CI [1.85, 2.93]) (Table 2). When stratifying by age at follow‐up, the risk of influenza hospitalization was highest below the age of 2 (overall rate <1 year 9.9 per 10,000 person‐years, 95% CI [8.7, 11.2]; 1 to <2 years 10.6 per 10 000 person‐years, 95% CI [9.4, 12.0]; 2 to <5 years 3.8 per 10,000 person‐years, 95% CI [3.4, 4.3]). The increased risk of influenza hospitalization for preterm born children compared with term children was similar across all age periods (<1 year aHR 2.24, 95% CI [1.44, 3.50]; 1 to <2 years aHR 2.26, 95% CI [1.58, 3.25]; 2 to <5 years aHR 2.47, 95% CI [1.66, 3.66]; Table 3).

**TABLE 2:** Association between gestational age category and influenza hospitalization before age 5 years

	No. of children	No. of person‐years at risk	No. of influenza cases	Rate per 10,000 py (95% CI)	Hazard ratio (95% CI)	
					Unadjusted	Adjusted^a^
Total number of children	238,628	1,180,599	754	6.4 [5.9, 6.9]		
Term (≥37 weeks)	223,542	1,107,171	653	5.9 [5.5, 6.4]	Ref	Ref
Preterm (<37 weeks)	15,086	73,427	101	13.8 [11.3, 16.7]	2.33 [1.88, 2.89]	2.33 [1.85, 2.93]
Extremely/very preterm (<32 weeks)	2145	9722	23	23.7 [15.7, 35.6]	4.00 [2.60, 6.17]	4.07 [2.63, 6.31]
Moderate/late preterm (32 to <37 weeks)	12,941	63,706	78	12.2 [9.8, 15.3]	2.08 [1.63, 2.65]	2.07 [1.60, 2.68]

Abbreviation: CI, confidence interval.

^a^ Adjusted for season of birth, sex, maternal age, multiple birth, maternal smoking, birth order and parental educational level.

**TABLE 3:** Association between preterm birth and influenza hospitalization by age group

Age group		No. of person‐years at risk	No. of influenza cases	Rate per 10,000 py (95% CI)	Hazard ratio (95% CI)	
					Unadjusted	Adjusted^a^
<1 year	All	237,929	235	9.9 [8.7, 11.2]		
	Term (≥37 weeks)	223,106	205	9.2 [8.0, 10.5]	Ref	Ref
	Preterm (<37 weeks)	14,823	30	20.2 [14.2, 28.9]	2.20 [1.47, 3.30]	2.24 [1.44, 3.50]
1 to <2 years	All	237,000	252	10.6 [9.4, 12.0]		
	Term (≥37 weeks)	222,255	216	9.7 [8.5, 11.1]	Ref	Ref
	Preterm (<37 weeks)	14,745	36	24.4 [17.6, 33.8]	2.51 [1.74, 3.63]	2.26 [1.58, 3.25]
2 to <5 years	All	705,670	267	3.8 [3.4, 4.3]		
	Term (≥37 weeks)	661,811	232	3.5 [3.1, 4.0]	Ref	Ref
	Preterm (<37 weeks)	43,860	35	8.0 [5.7, 11.1]	2.28 [1.60, 3.25]	2.47 [1.66, 3.66]

Abbreviation: CI, confidence interval.

^a^ Adjusted for season of birth, sex, maternal age, multiple birth, maternal smoking, birth order, and parental educational level.

Children born moderately or late preterm (32–37 weeks) had a higher risk of hospitalization compared with those born at term (aHR 2.07, 95% CI [1.60, 2.68]), and those born <32 weeks (extremely or very preterm) had a fourfold higher risk of hospital admission for influenza (aHR 4.07, 95% CI [2.63, 6.31]) (Table 2) compared with children born at term.

When stratifying gestational age at birth into 1 or 2‐week categories, the risk of hospital admission for influenza increased with decreasing gestational age (Table 4). Compared with children born at 40–41 weeks, children born late preterm (35–36 weeks, *n* = 8854) had a higher risk of hospitalization (aHR 2.18, 95% CI [1.59, 3.00]). Among children born at term, 42530 (19%) were born at 37 or 38 weeks (defined as ‘early term’). Compared with children born at 40–41 weeks, these early term children also had an elevated risk of hospital admission for influenza (aHR for 37 weeks 1.89, 95% CI [1.43–2.50]; aHR for 38 weeks 1.43, 95% CI [1.15, 1.78]). The cumulative incidence of hospital admission for influenza for children born post‐term (≥42 weeks, *n* = 13,702) was slightly higher (6.5 per 10,000 person‐years compared with 5.3 for children born 40–41 weeks), though this difference was not significant (aHR of 1.25, 95% [0.91, 1.72]).

**TABLE 4:** Association between gestation (by week) and influenza hospitalization before age 5 years

	No. of children	No. of person‐years at risk	No. of influenza cases	Rate per 10,000 py (95% CI)	Hazard ratio (95% CI)	
					Unadjusted	Adjusted^a^
<33 weeks	2966	13,708	29	21. 2 [14.7, 30.4]	4.01 [2.68, 6.02]	4.25 [2.83, 6.37]
33–34 weeks	3266	16,051	23	14.3 [9.5, 21.6]	2.73 [1.76, 4.24]	2.84 [1.82, 4.42]
35–36 weeks	8854	43,669	49	11.2 [8.5, 14.8]	2.13 [1.57, 2.90]	2.18 [1.59, 3.00]
37 weeks	12,453	61,599	61	9.9 [7.7, 12.7]	1.88 [1.42, 2.49]	1.89 [1.43, 2.50]
38 weeks	30,077	148,847	114	7.7 [6.4, 9.2]	1.46 [1.17, 1.81]	1.43 [1.15, 1.78]
39 weeks	54,373	269,338	140	5.2 [4.4, 6.1]	0.99 [0.81, 1.21]	0.98 [0.80, 1.20]
40–41 weeks	112,937	559,572	294	5.3 [4.7, 5.9]	Ref	Ref
42+ weeks	13,702	67,815	44	6.5 [4.8, 8.7]	1.23 [0.90, 1.69]	1.25 [0.91, 1.72]

Abbreviation: CI, confidence interval.

^a^ Adjusted for season of birth, sex, maternal age, multiple birth, maternal smoking, birth order and parental educational level.

Preterm birth is common in multiple pregnancies. To exclude the possibility that multiple births were driving the observed associations between preterm birth and influenza, we repeated our main analysis after excluding multiple births. The results were almost identical to the results with multiples included (aHR for singletons only: 2.36; 95% CI [1.86, 2.99], Table S3).

## 4 DISCUSSION

Our results show that children who were born preterm had a higher risk of hospitalization with influenza up to age five. The increased risk was apparent also for children born early term, and the risk increased with each week of shorter gestational age. Children born extremely preterm (<32 weeks) had a more than fourfold risk of hospitalization with influenza in the first 5 years of life compared with those born at term.

In our study, 6.3% of the children were born preterm (<37 weeks), corresponding to 5.6% of pregnancies, as many multiples are born preterm. This is similar to rates in other Nordic countries, which have preterm rates of 5.6% (Sweden, 2001),^32^ 6.3% (Denmark, 2004)^17^ and 5.2% (Finland, 2001–2005).^33^

Our results support previous studies finding that lower gestational age is associated with an increased risk of hospital admissions due to respiratory infections, and that risk of infection increase with lower gestational age.^19^, ^34^ A systematic review investigated a range of risk factors for children hospitalized with influenza and concluded that prematurity was one of the most important risk factors.^35^ However, five of the seven studies included in this review did not define prematurity in terms of gestational age, and they were unable to assess the risk beyond the first 2 years of life. With the lack of supporting evidence, preterm children are still not defined as a risk group and prioritized for influenza vaccination.

Increased susceptibility to severe influenza could partly be explained by sequelae and comorbidities associated with preterm birth.^22^ Neonates and preterm infants even more so, have an immature immune system in the first months of life. Foetal lungs develop gradually, and preterm birth interrupts the normal maturing process, but also additional factors contribute to the increased susceptibility for infections.^36^, ^37^ An estimated 40% of children born extremely preterm develop sequelae including bronco‐pulmonary dysplasia (BPD),^38^ which is observed to cause reduced respiratory function that persist into late adolescence and adulthood.^39^, ^40^ Increasingly lower gestational age is associated with reduced lung function.^41^

Previous research primarily reports an increased risk for severe influenza for children born moderately to extremely preterm. These studies lacked sufficient detail to assess the association between the extent of prematurity and risk of hospital admission, and the need for further studies has been emphasized.^35^ Some researchers have addressed overall respiratory morbidity among those born moderately/late preterm and early term. Findings support an increased risk for respiratory hospitalization for those both born late preterm (35–36 weeks) and born even early term (37–38 weeks), but these studies have not addressed risk of influenza hospitalizations in particular.^42^, ^43^, ^44^, ^45^, ^46^ Sequelae (such as reduced lung function) is known to increase the risk of severe influenza, but is only present in a minority of preterm children. The majority, also those born extremely preterm, have no known bronchopulmonary dysplasia later in life.^38^ The increased risk of influenza hospitalization we found in this study among the early term and late preterm is therefore not likely to be explained by long‐term respiratory‐related sequela.

All registry‐based studies are prone to some misclassification. According to the ICD‐10 diagnostic guidelines, laboratory testing for influenza was not required for all the ICD‐10 diagnoses we used to define hospitalizations with influenza. In the absence of laboratory confirmation, recording of an influenza diagnosis depends on the clinician's judgement of the clinical symptoms and patient history and therefore may be incorrect. However, 83% of the influenza hospitalizations included in our study were associated with ICD‐10 J09 or J10 diagnosis, which requires laboratory confirmation. Previous studies have found that ICD‐10 data underestimate rather than overestimate the numbers of influenza cases, with a high specificity for influenza diagnoses.^47^ Data on laboratory testing for seasonal influenza is not recorded in national registries, and we did not have information on laboratory testing in our study. We aimed to reduce potential misclassification of influenza diagnoses by excluding hospitalizations outside the influenza surveillance period. It may be that children who were born preterm and are admitted to hospitals with influenza symptoms are disproportionately likely to be tested and diagnosed with influenza, causing a potential inflation of association. However, we believe that preterm birth is less likely to be considered a key patient characteristic when treating children for influenza beyond the infant period and also that most children with symptoms will have been tested (irrespective of preterm status).

We were able to stratify preterm births into extremely/very preterm (<32 weeks) and moderately/late preterm. However, we were unable to further stratify the former category into extremely or very preterm for anything other than descriptive analysis due to small cell counts.

Although preterm birth is highly correlated with low birth weight, our aim was to address the total effect of being born preterm (which includes lower birth weight) rather than assessing the independent effects of gestational age and birth weight, which are difficult to disentangle.^48^

We did not include data on the vaccination status of the child or the mother in our study. However, in Norway, children in general are not classified as a priority group for influenza vaccination. Although children with chronic diseases are recommended for the annual influenza vaccine, survey data from Norway indicate that vaccination coverage is far from optimal, with only around 39% coverage in high risk groups (all ages) in 2018–2019.^49^ Therefore, it is likely that only a small proportion of children in Norway have received the annual influenza vaccination during the study period, and it is likely that children who received vaccination had higher underlying susceptibility to infection due to other risk factors. Equally, some infants may have received some protection in the first months after birth via maternal immunization during the in utero period. However, uptake of influenza vaccination during pregnancy in Norway is low,^50^ and protection conferred by maternal vaccination is not likely to persist past the infant period.^51^

## 5 CONCLUSION

Children born preterm were at increased risk of hospitalization with influenza infection during their first 5 years of life. This risk increased with decreasing gestational age at birth and was fourfold for children born before 32 weeks of gestation. The risk of hospitalization for influenza was also higher in children born at an early term gestation compared with children born in gestational Weeks 40 and 41. Our results indicate that children who were born preterm are at higher risk for severe influenza and could be considered as a risk group when evaluating for recommendations for influenza vaccination. In settings where children are already included in routine influenza vaccination programmes, vaccine hesitancy is still an obstacle,^52^ and efforts should be made to communicate the importance of influenza vaccination in this high‐risk group of children.

## AUTHOR CONTRIBUTIONS

**Siri E Håberg:** Conceptualization; formal analysis; funding acquisition; methodology; project administration; resources; supervision; validation. **Laura Oakley:** Conceptualization; data curation; formal analysis; methodology; supervision; validation; visualization.

## CONFLICT OF INTEREST

The authors have no conflict of interest to disclose.

## FUNDING INFORMATION

Funded/supported by the Norwegian Institute of Public Health and by the Norwegian Research Council's Centre for Excellence (scheme #262700).

## ETHICS STATEMENT

The study was approved the Regional Ethics Committee of South‐Eastern Norway, #2010/2583 which waived the need for consent.

No material has been reproduced from other sources.

### PEER REVIEW

The peer review history for this article is available at https://publons.com/publon/10.1111/irv.12908.

## Supporting information


**Table S1.** Data sources
Table S2. Influenza hospitalization before age 5 years, by gestational age at birth, among children born between January 1, 2008 and December 31, 2011 in Norway (N = 238 628).
Table S3. Associations between preterm birth and influenza hospitalization before age 5 years, excluding 7872 multiple births
Figure S1. Study flowchart

## Data Availability

Data used in this manuscript are available for other researchers by standard application to the registries. Disclaimer: Data from the Norwegian Patient Registry have been used in this publication. The interpretation and reporting of these data are the sole responsibility of the authors, and no endorsement by the Norwegian Patient Registry is intended nor should be inferred.
