# Supplementary material for: Influenza hospitalizations during childhood in children born preterm
Source: Influenza Other Respir Viruses. 2021 Sep 14;16(2):247–54. doi: 10.1111/irv.12908 (PMC8818812; doi:10.1111/irv.12908)
Supplement: Supplementary file 1 — Table S1. Data sources Table S2. Influenza hospitalization before age 5 years, by gestational age at birth, among children born between January 1, 2008 and December 31, 2011 in Norway (N = 238 628). Table S3. Associations between preterm birth and influenza hospitalization before age 5 years, excluding 7872 multiple births Figure S1. Study flowchart [file IRV-16-247-s001.docx]

**+SUPPLEMENTARY TABLES AND FIGURES**

**Table S1. Data sources**

| **Variable type** | **Variable name** | **Source** | **Categorization** |
| --- | --- | --- | --- |
| **Exposure** | **Preterm birth** | The Medical Birth Registry of Norway (MBRN)  *Gestational age based on best available estimate (98% ultrasound, 2% LMP)* | Extremely or very preterm: <32 completed weeks  Moderately or late preterm: 32 to <37 completed weeks  Term: ≥37 completed weeks  Preterm: <37 completed weeks |
| **Outcome** | **Influenza hospital admission** | The Norwegian Patient Registry (NPR)  Hospital admission with any of the following ICD-10 codes: J09 “Influenza due to identified zoonotic or pandemic influenza virus”, J10 “Influenza due to identified seasonal influenza virus” or J11 “Influenza, virus not identified”  *Admissions during the yearly influenza surveillance periods only (May to October)* | Yes/No |
| **Covariates** | **Maternal age at delivery** | MBRN | <20 years  20-24 years  25-29 years  30-34 years  35-39 years  ≥40 years |
|  | **Birth order** | MBRN | 1: first pregnancy  ≥2: second or subsequent pregnancy |
|  | **Multiple birth** | MBRN | Yes/No |
|  | **Maternal smoking** | MBRN  Maternal smoking recorded at antenatal care visit | Yes/No/Information declined |
|  | **Season of birth** | MBRN | Winter: December, January, February  Spring: March, April, May  Summer: June, July, August  Autumn: September, October, November |
|  | **Parental college/ university education** | Statistics Norway  Education status for mother and father for 2013 | Yes: at least one parent (mother or father, or both) has a college or university education  No: neither parent has a college or university education |

**Table S2. Influenza hospitalization before age 5 years, by gestational age at birth, among children born between January 1, 2008 and December 31, 2011 in Norway (N = 238 628).**

| Gestational age at birth(week) | No. of children | No. of children with influenza hospitalization | |
| --- | --- | --- | --- |
|  |  | **n** | (**%**) |
| <28 | 585 | 10 | (1.71) |
| 28-29 | 563 | 4 | (0.71) |
| 30 | 435 | 4 | (0.92) |
| 31 | 562 | 5 | (0.89) |
| 32 | 821 | 6 | (0.73) |
| 33 | 1 239 | 11 | (0.89) |
| 34 | 2 027 | 12 | (0.59) |
| 35 | 3 117 | 18 | (0.58) |
| 36 | 5 737 | 31 | (0.54) |
| 37 | 12 453 | 61 | (0.49) |
| 38 | 30 077 | 114 | (0.38) |
| 39 | 54 373 | 140 | (0.26) |
| 40 | 66 409 | 158 | (0.24) |
| 41 | 46 528 | 136 | (0.29) |
| ≥42 | 13 702 | 44 | (0.32) |
| TOTAL | 238 628 | 754 | (0.32) |

**Table S3. Associations between preterm birth and influenza hospitalization before age 5 years, excluding 7872 multiple births**

|  | No. of children | No. of person-years at risk | No. of influenza cases | Rate per 10 000 py  (95% CI) | Hazard ratio (95% CI) | |
| --- | --- | --- | --- | --- | --- | --- |
|  |  |  |  |  | **Unadjusted** | **Adjusted*** |
| Total number of children | 230 756 | 1 142 010 | 717 | 6.3 (5.8-6.8) |  |  |
| Term  (≥37 weeks) | 219 428 | 1 086 847 | 640 | 5.9  (5.5-6.4) | ref | ref |
| Preterm  (<37 weeks) | 11 328 | 55 163 | 77 | 14.0 (11.2-17.4) | 2.37  (1.87-3.00) | 2.36  (1.86-2.99) |

*Adjusted for season of birth, sex, maternal age, maternal smoking, birth order, and parental educational level

**Figure S1. Study flowchart**

238 628 eligible children

15 086 children born preterm

(<37 weeks)

245 281 children born between January 1, 2008 and Dec 31, 2011

2 482 children excluded due to missing mother ID, poor linkages, missing or implausible gestational age, stillborn, or died on day of birth

223 542 children born at term

(≥37 weeks)

12 941 born moderate/late preterm (32-<37 weeks)

4 171 children excluded due to missing covariate information

585 born extremely preterm

(<28 weeks)

1 560 born very preterm

(28-<32 weeks)
